# Stimuli-Responsive Nanomaterials for Application in Antitumor Therapy and Drug Delivery

**DOI:** 10.3390/pharmaceutics12070630

**Published:** 2020-07-04

**Authors:** Son H. Pham, Yonghyun Choi, Jonghoon Choi

**Affiliations:** School of Integrative Engineering, Chung-Ang University, Seoul 06974, Korea; sonpham11.bka@gmail.com (S.H.P.); dydgus5057@gmail.com (Y.C.)

**Keywords:** nanoparticles, stimuli-responsive, drug delivery, nanomedicine

## Abstract

The new era of nanotechnology has produced advanced nanomaterials applicable to various fields of medicine, including diagnostic bio-imaging, chemotherapy, targeted drug delivery, and biosensors. Various materials are formed into nanoparticles, such as gold nanomaterials, carbon quantum dots, and liposomes. The nanomaterials have been functionalized and widely used because they are biocompatible and easy to design and prepare. This review mainly focuses on nanomaterials responsive to the external stimuli used in drug-delivery systems. To overcome the drawbacks of conventional therapeutics to a tumor, the dual- and multi-responsive behaviors of nanoparticles have been harnessed to improve efficiency from a drug delivery point of view. Issues and future research related to these nanomaterial-based stimuli sensitivities and the scope of stimuli-responsive systems for nanomedicine applications are discussed.

## 1. Introduction

Nanotechnology research has had a significant impact on the field of medicine. Particularly, nanomaterials, such as liposomes, dendrimers, polymers, metallic nanoparticles (NPs), and nanogels, are currently being developed and used in various biomedical applications. These nanomaterial systems play an important role not only in sensing, biomedical imaging, and diagnosis but also in drug delivery. These smart carrier systems have been widely developed because they are responsive to various exogenous (e.g., pH, temperature, light, and ultrasound) and endogenous (e.g., redox potential and enzyme presence) stimuli.

A variety of nanoparticles are suitable for drug delivery; thus, the development of nanoparticle drug-delivery systems with stimuli-responsive properties has gained considerable attention [1,2,3,4]. There are two approaches for designing stimuli-responsive drug-delivery nanoparticles that efficiently accumulate at the tumor site, respond to intratumoral or external stimuli, and penetrate tumor cells. One of these approaches involves endogenous stimuli (i.e., enzymes, ions, and redox potential), which are mainly unique among tumor regions and can effectively enhance drug release [5,6]. This approach requires the careful selection of materials that can form effective nanocarriers, respond to specific endogenous stimuli, and release enclosed drugs. In the second approach, exogenous physical stimuli (i.e., temperature, light, electricity, magnetic fields, and ultrasound) are applied to the target tissue [7]. These exogenous stimuli can disrupt the structure of specifically designed nanocarriers and cause them to release a drug in the desired tissue. One benefit of a stimuli-responsive drug-delivery system is that it can prevent premature drug release, which is a common problem with traditional drug-delivery systems [8,9]. Therefore, research trends have moved toward stimuli-responsive drug-delivery systems that rely on a combination of two or more stimuli-responsive systems to increase targeting efficiency [7,10,11]. However, exogenous stimuli-responsive materials have a few limitations. It is challenging to find exogenous stimuli that can penetrate the human tissue and precisely reach the tumor site. Additionally, endogenous stimuli that will control the release specifically at the tumor site in an appropriate time and dosage are hard to find.

**Figure 1:**
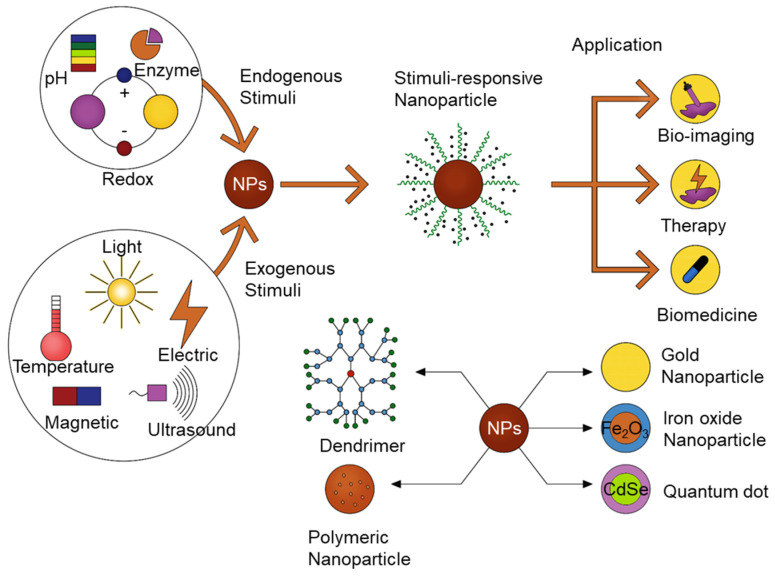
Schematic diagram of stimuli-responsive nanomaterials used for bioimaging, therapy, and triggered drug release.

In this review, endogenous and exogenous stimuli, along with dual- and multi-stimuli-responsive drug delivery, are discussed (Figure 1). This review would provide a summary of current research on various stimuli and responding nanoparticles as well as their up-to-date application. While there were several reports on stimuli-responsive block copolymer carriers, this review would not limit the scope only on the polymeric nanocarriers. The state-of-art nanosized materials responding to external and internal stimuli would be discussed.

## 2. Endogenous Stimuli-Responsive Systems

### 2.1. Redox-Responsive Systems

The redox potential of tumor cells is 100- to 1000-fold higher than that of other cells [12]. Glutathione (GSH) is an important antioxidant that can inhibit the reactive oxygen species (ROS) produced in cellular components. The redox potential difference between the extracellular (oxidative) and intracellular (reductive) spaces is associated with the extracellular and intracellular GSH concentrations [13].

The development of drug-delivery systems based on GSH-responsive assemblies has gained increasing attention. GSH can destabilize disulfide linkages in nanoparticles in a redox-mediated manner. Block copolymers contain disulfide linkages between hydrophilic and hydrophobic blocks that are GSH-responsive. This enables the formation of micelles, which are also known as ‘shell-sheddable’ micelles. When these micelles interact with GSH, they become destabilized and release therapeutic materials [14,15].

High concentrations of reducing materials in intracellular compartments help maintain polymers with disulfide linkages; however, polymers containing hydrophobic blocks (i.e., polypropylene sulfide [16,17,18]) are sensitive to the oxidative environments created by many disease states. Pioneering works by Tirelli et al., showed polysulfides nanocarriers that could respond to the presence of oxidants and their use for immune modulating applications [16,17,18]. Hydrophobic parts in polymers were also used for the oxidation-induced solubility variations in a selenium block copolymer. Spherical micelles were generated from copolymers in an aqueous medium with hydrophobic selenium cores. The oxidation of selenium to selenoxide [19] using hydrogen peroxide led to a phase transition from hydrophobic to hydrophilic causing the micelles to disassemble [19].

By adding reducing agents, spherical micelles were reproduced, and it showed that these selenium-containing nanocapsules were responsive to oxidizing and reducing conditions.

Disulfide linkages have been frequently used to design nanocarriers. For example, redox-responsive capsules were prepared by combining poly (*N*-vinyl pyrrolidone) (PVPON), poly (methyl methacrylate) (PMAA), and a disulfide cross-linker that could deliver plasmid DNA and doxorubicin [20]. One concern with this technology is its stability and premature drug release since cysteine and GSH are present in the extracellular compartment. This problem may be solved using manifold disulfide linkages created by adjusting the number of disulfide cross-links [21].

### 2.2. pH Stimuli-Responsive Systems

The rapid proliferation of cancer cells triggers glycolysis and reduces a pH in the tumor microenvironment that would contribute to a controlled drug release [22]. When pH-sensitive nanoparticles are exposed to these acidic regions, the chemical structure of certain nanoparticles changes and allows the release of their drug payload [23].

Both organic and inorganic materials are used to making pH stimuli-responsive nanoparticles [24]. Dendritic polymers have been widely used in pH-sensitive systems because their solubility, conformation, and volume can be easily manipulated. Polyethylene glycol is applied to the surface of dendritic polymers to alter size, structure, and biocompatibility [25]. This type of polymer can enhance drug loading and solubility and increase the accumulation of drugs in tumor tissues via enhanced permeability and retention. A higher-efficacy cancer treatment could be obtained by coupling antitumor drug molecules to dendrimers via a hydrazone bond formation [26,27].

Nanoparticles containing pH-sensitive precursor drugs that can deliver hydrophobic anticancer drug combinations have been successfully developed [28]. For example, PEG nanoparticles loaded with curcumin (CUR) and doxorubicin (DOX) were combined with transferrin (Tf), resulting in nanocomplexes. The simultaneous release of CUR and DOX was significantly accelerated under mildly acidic environments. Some studies have shown that 79.2% and 57.6% of DOX is released from NPs within 24 h at pH values of 5.0 and 7.4, respectively. Tf attached nanocomplexes showed advantages as drug-delivery carriers in their well-defined size and high drug encapsulation yields [29].

One interesting drug-delivery strategy involves self-assembled hyaluronic acid nanoparticles that use calcium phosphate to form hydroxyapatite nanoparticles loaded with DOX. When exposed to a low pH, the minerals in this system dissolve, causing drug release at a specific tumor site [30].

### 2.3. Enzyme Stimuli-Responsive Systems

Some nanomaterials, including polymers, phosphodiester, and inorganic materials, have been used to construct enzyme-responsive drug-delivery systems. Ester bonds or the peptide structure of stimuli-responsive nanocarriers can be degraded by various enzymes specific to inflammation of the tumor location. This degradation releases the drug payload of these nanocarriers at targeted sites [31,32,33].

Enzyme-responsive systems offer unique features, such as biorecognition, process efficiency, sensitivity, selectivity, and catalytic efficacy. In these systems, the drug payload is released upon enzyme-assisted degradation of polymeric moieties. Typically, two classes of enzymes serve as triggers in enzyme-responsive drug delivery: Proteases (or peptidases) and phospholipases [34]. Proteases are particularly advantageous in fabricating these drug-delivery systems because they are often over-expressed during infection, cancer, and inflammation. Trypsin, one of the essential digestive proteinases, helps control exocrine pancreatic secretion, which is involved in the stimulation of several other digestive enzymes [35]. Phospholipase A_2_ (PLA_2_) is gaining much attention as a therapeutic target due to its upregulation in the tumor microenvironment. For instance, Mahdi Ghavami et al. prepared the phospholipase sensitive liposome (PSL) as a drug-delivery system, in which liposome degradation is caused by a tumor cell-secreted phospholipase A_2_ (sPLA_2_). sPLA_2_ is responsible for triggering the release of active PNA (peptide nucleic acid) as an antisense agent from PSL. The results showed that >80% of phospholipase was utilized to activate the PNA release. Thus, phospholipase may act usefully as a triggered agent to release drugs from nano-delivery platforms [36].

An enzyme-responsive nanomaterial adapting an HPMA triblock copolymer was developed [37]. The polymer was synthesized and self-assembled into nanoparticles with diameters of approximately 85 nm. The high molecular weight (92 kDa) copolymeric system was degraded into small segments with molecular weights less than 50 kDa and specifically released the anticancer drug paclitaxel in the cancer microenvironment. This type of system may lead to a simultaneous treatment and magnetic resonance imaging of breast cancer.

### 2.4. microRNA Responsive Systems

There are recent developments in microRNA responsive systems that are employing nanomaterials to deliver microRNA (miRNA) specifically to the target. miRNA is defined as a class of small non-coding RNAs, including short nucleotide sequences, 20–24 mer nucleotides that can serve as key regulators of gene expression [38]. Previous studies have described the influence of miRNA on the onset and progression of diseases, including cancer, cardiovascular diseases, and other pathologies. Such studies focus on the changes in miRNA expression between normal and tumor regions, resulting in miRNA being considered as therapeutic agents. Although there have been some advantageous characteristics in using miRNA for therapeutic purposes, there are still obstacles remaining for the successful delivery of miRNA. The limitation includes the lack of stability of miRNA in cellular environments, poor targeting ability, high toxicity, inability to deliver sufficient miRNAs to the desired tissues, and off-target effects of naked miRNA-based agents [39]. To improve stability, several strategies involving the chemical modification of miRNA have been investigated based on utilizing ribose 2′- OH groups, locked nucleic acids, backbone modifications, and peptide nucleic acids [40]. The inefficient and non-specific delivery of miRNAs to cells, however, remains as a challenge.

The efficiency of delivering miRNA to the target has been significantly improved by loading miRNA with nanoparticles (NPs) protecting the miRNA from the external microenvironment, thereby reducing degradation and enhancing circulation time as well [41,42]. Nanocarriers that are biocompatible and require simple manufacturing were employed.

Gold nanoparticles (AuNPs) have gained much attention as nucleic acid delivery nanocarriers by their biocompatibility, facile synthesis, and tunable size and shape [43,44]. The surface of AuNPs is modified with thiol or amino groups to enable miRNA entrapment. Wang et al. have developed a composite of miRNA-124-5p and AuNPs to combine gene therapy and photothermal therapy for the effective killing of cancer cells. The release of miRNA-124-5p could be achieved by the cleavage of cystamine when the AuNPs enter the cytoplasm of tumor cells through endocytosis [45]. Ekin et al. also have used AuNPs to deliver the miRNA-145 into prostate or breast cancer cells [46].

Hyaluronic acid (HA), which is a highly hydrophilic natural polysaccharide, has been used to link PEI and PEG to a controlled-release miRNA and/or DOX [47]. Liang et al. have successfully synthesized HA-NPs loaded with miRNA-145 to target colon cancer cells through their overexpressed CD44 receptors [48]. In addition, the use of synthetic polymer NPs for nucleic acid delivery has also been investigated. Liu et al. have prepared PEG-peptide-PCL NPs to deliver miRNA-200c and docetaxel [49]. Wang et al. have produced PLGA-PEI NPs to carry miRNA-542-3p and DOX in breast cancer cells. An HA-decorated PEI-PLGA nanoparticle system increased both drug uptake and cytotoxicity in MDA-MB-231 cells compared to MCF-7 cells that express lower CD44 levels [47].

## 3. Exogenous Stimuli-Responsive Systems

Exogenous stimuli-responsive systems have the potential to overcome interpatient variability. In these systems, drug release can be precisely controlled by external factors. Exogenous physical stimuli mainly include temperature, light, magnetic fields, and ultrasound, which can be applied directly to the tissue of interest to stimulate drug release [50,51,52,53].

The main advantage of most external stimuli-responsive systems is that drugs can be controlled—releasing at the desired site, which minimizes off-target effects in the surrounding healthy tissue. However, some treatments that involve injecting material followed by activation with ultrasonic waves, advanced light sources, or strong magnetic fields may not always be practical or cost-effective. Another problem with exogenous stimuli is the depth of penetration needed to cause a drug-delivery system to release its payload [54]. To address this issue, long wavelengths of light or two-photon strategies are employed to expand the types of tissues that can be treated by light (e.g., a near-infrared laser) [55].

### 3.1. Temperature Stimuli-Responsive Systems

Temperature- or thermo-responsive systems are one of the most widely explored exogenous stimuli-responsive systems for treatment and diagnostic applications [1,56]. The nanocarriers responding to an external temperature change over the critical solution temperature can release their drug payload when the polymer chain dehydrates. Generally, diseased/tumor tissues have a higher temperature (40–42 °C) than normal tissues (37 °C) [54]. Therefore, thermo-responsive drug carriers retain their drug payload at normal temperatures and release it when exposed to the elevated temperatures of the diseased/tumor tissue.

There are two strategies for a thermo-responsive drug release. One strategy involves drug carriers that are designed for a burst drug release when exposed to high temperatures. For example, polycaprolactone(*N*-isopropyl acrylamide) (PNIPAM) conjugated carbon nanomaterials were prepared with drug molecules to treat tumors [56]. These drug-loaded NPs were thermo-responsive and released more substantial amounts of drugs at a higher temperature (40 °C). Moreover, the cell viability (20%) at 40 °C was significantly lower than that at 37 °C (40%) upon treatment with the drug-loaded nanoparticles.

The second approach involves drug carriers that are designed for a burst drug release when exposed to higher temperatures caused by an external stimulus. The external stimulus causes a sensitive drug carrier agent to produce heat, which alters temperature-sensitive materials in the drug carrier, leading to a burst drug release at the target site [57]. An amphiphilic di-block copolymer prepared with temperature-responsive materials including polypyrrole (PPy) was loaded with antitumor drug molecules [58]. Upon near-infrared (NIR) absorption, PPy produced heat because of the photothermal effect, and the increase in temperature promoted a drug release from the micelles. The release was caused by the swelling of the thermo-sensitive polymer via hydrophobic to hydrophilic conversion [58].

Thermo-responsive polymers are critical components of these systems because they can respond to temperature changes. Thermo-responsive materials have two main characteristics: The lower CST (critical solution temperature) (LCST) and the upper CST (UCST) [59]. A decrease in temperature below the LCST leads to swelling owing to increased hydrophilicity, and an increase in temperature above the UCST leads to increased hydrophilicity. Changes in hydrophilicity control the swelling behavior of the drug carriers and allow for the fine-tuning of drug release. For instance, PNIPAM is a widely-used building block for thermo-responsive carriers. Its solubility in water differs based on the temperature variability from its LCST. Above their LCST, PNIPAM coils transform into globules that are water-insoluble; thus, a drug release can be controlled due to the dominance of hydrophobic interactions [60]. Hydrophilic PNIPAM below its LCST was utilized for higher drug loading, and its hydrophobic behavior above its LCST was useful for cell attachment and a sustained release. Moreover, its LCST can be modified by changing the ratio of the hydrophilic and hydrophobic parts.

### 3.2. Photo/Light-Responsive Systems

Specific wavelengths of light (i.e., ultraviolet, visible, and near-infrared light) can change the stability or structural degradation of responsive nanomaterials in light-responsive drug-delivery systems to release drugs at precise locations.

Owing to a low penetration capability, visible and ultraviolet light are not suitable for in vivo therapeutic applications. However, a NIR-responsive system is a promising technique that uses light to control drug release because it displays better penetration and limited tissue damage [61]. There are three different drug-release mechanisms used in NIR-responsive systems: The photothermal effect, two-photon absorption (TPA), and upconverting nanoparticles (UCNP).

The photothermal effect involves the conversion of light to heat by a photothermal agent contained in the nanocarrier. This heat stimulates the heat-sensitive material and disrupts the nanostructure, breaks the hydrophobic and hydrophilic linkages, or creates a phase transition that leads to rapid drug release at the tumor site. Recently, Li et al. produced multiple nanostructured lipid carriers encapsulated by liposomes that were loaded with the hydrophilic drug AMD3100 and the hydrophobic NIR photothermal agent IR780 [62]. NIR light stimulated IR780 to produce heat, which then destabilized the liposomal membrane and caused drug release. IR780 also induced cytotoxic hyperthermia as a synergistic effect along with chemotherapy.

TPA relies on the excitation by two absorbed photons of identical or different frequencies [63]. For instance, Guardado-Alvarez et al. created mesoporous silica nanoparticles (MSNPs) with a disulfide-linked *β*-cyclodextrin cap [64]. The TPA based transducer provided an electron for the reduction of the disulfide linker. This caused the removal of the *β*-cyclodextrin cap, which led to drug release. In summary, TPA is a promising strategy for controlled drug delivery because a NIR laser has high spatial and temporal resolutions, deep tissue penetration, and low scattering losses. However, this technique requires a focal pulsed laser with high energy density to treat a small infection area. Therefore, this method is not ideal for in vivo experiments.

The UCNP technique can convert NIR light to UV light to activate high energy light-sensitive materials [65]. To illustrate, Xiang et al. synthesized and coated UCNPs with an amphiphilic di-block copolymer containing a UV-sensitive hydrophobic layer made of poly(4,5-dimethoxy-2-nitrobenzyl methacrylate) and an outer hydrophilic layer made of poly(methoxy polyethylene glycol monomethacrylate) [61]. When exposed to NIR irradiation (908 nm), a copolymer absorbed the UV light and produced an imbalance in the hydrophilic-hydrophobic equilibrium that caused the micelle structure to change and release the encapsulated drug [61]. The poly (4,5-dimethoxy-2-nitrobenzyl methacrylate) (PNB) absorbed UV light and transformed a hydrophobic polymer block into a hydrophilic block and resulted in the dissolution of a di-block polymer and releasing of the drug molecules. Some NIR-responsive systems use a photosensitizer to create a synergistic effect by producing ROS in addition to chemotherapy [66,67].

Huang et al. reported that ultrathin InSe nanosheets were efficient NIR-II-responsive drug-release agents [68]. PEGylated InSe nanosheets exhibited excellent biocompatibility and physiological stability, NIR-II photothermal conversion efficiency of 39.5%, and a high DOX-loading capacity of 93.6% (*w*/*w*). The release rate of DOX was accelerated under NIR-II irradiation owing to the on/off switching stimulus results. This indicates that NIR-II-induced local hyperthermia can release DOX from InSe-DOX in a controlled manner and at a predetermined time. DOX release can reach a maximum under acidic conditions with irradiation. Thus, the pH-responsiveness, NIR-II sensitivity, and switchable release characteristics of the InSe nanoplatform make it advantageous as a targeted chemotherapy delivery method [68].

### 3.3. Magnetic-Responsive Systems

Magnetic systems are commonly used for body imaging (e.g., MRI) and a controllable drug release through external stimuli [69]. They are widely applicable because they are biocompatible, biodegradable, easy to synthesize as a co-precipitate or micro-emulsion, and simple to modify and functionalize for specific applications. Magnetic nanoparticles (MNPs) are small, have a large specific surface area, and can easily reach desired locations [69]. Therefore, MNPs are promising as a potential drug-delivery system.

The basic mechanism of magnetic stimuli-responsive drug-release systems involves their ability to generate heat via an alternate magnetic frequency. MNPs can function as transducers to convert hysteresis loss and Néel relaxation to heat under an alternating current magnetic field. Two treatment mechanisms based on magnetic-responsive systems have been reported. One mechanism involves magnetic field-induced hyperthermia [70], and the other involves magnetic field-guided drug targeting [71].

Hyperthermia-based magnetic systems have been explored for drug delivery and tumor inhibition applications due to their magnetic responses [72]. Thirunavukkarasu et al. synthesized superparamagnetic iron oxide (Fe_3_O_4_) nanoparticles (SPIONs) for theranostic purposes [70]. They loaded SPIONs and DOX in a poly (lactic-co-glycolic acid) (PLGA) matrix, which responds to the heat produced by the SPIONs under magnetic field exposure, releasing DOX. In vitro studies showed the temperature sensitivity of the PLGA matrix with a ~39% and ~57% drug release observed at 37 and 45 °C, respectively. Iron oxide nanoparticles under the external application of AMF made a change in the solution temperature, leading to the transition of the PLGA polymer matrix and subsequently releasing the drug molecules. In another report, Wang et al. produced an implantable magnetic chitosan hydrogel loaded with hydrophobic (rifampicin) and hydrophilic (adriamycin) drugs [73]. The implantable magnetic chitosan hydrogel responded to an external low frequency alternating magnetic field and released the drugs in a pulsatile manner without producing magnetic hyperthermia. This system was designed to actively control the drug release and prevent post-surgical infections [73].

### 3.4. Ultrasound-Responsive Systems

Ultrasound technology has been used in therapeutic applications, such as imaging-guided drug delivery, because it is safe, penetrates tissues, is non-invasive, and has better spatiotemporal control since it can precisely focus on a target area. The thermal and mechanical effects, as well as the radiation forces generated by ultrasound waves, are responsible for stimulating a carrier drug release [74].

Paris et al. synthesized mesoporous silica nanoparticles (MSNPs) PEGylated by the thermo-responsive linker (4,4′-azobis (4-cyanovaleric acid)) [75]. When an external ultrasound was applied, it induced heat, cleaved the thermo-responsive linker, and led to the drug release. Papa et al. manufactured injectable, ultrasound-responsive PLGA nanoparticle aggregates [76]. After irradiation with a local and low-energy ultrasound, nanoparticle aggregates loaded with DOX were degraded into PLGA nanoparticles, which then entered the tumor via the enhanced permeability and retention effects. Thus, the drugs were released and accumulated into the tumor sites [76].

### 3.5. Electric Field-Response Systems

Electric fields are among the various physical triggers that have helped increase the efficiency of drug therapy. High-intensity exogenous electric fields can directly influence the permeability of cellular membranes, be used as a stimulus for drug delivery, or serve as therapeutic tools for wound healing and the restoration of tissue integrity.

The mechanism underlying this approach involves the reduction of the conducting polymer when a potential is applied. This causes the drug that is incorporated into the polymer to be discharged into the surrounding environment by electrostatic repulsion. The rate of drug release depends on the polymer’s morphology (including its density) or electrochemical properties or the characteristics of the solution surrounding the drug-delivery system (e.g., pH, temperature, and the addition of the dopants). Recently, Neumann et al. reported a strategy for electro-responsive drug delivery [77]. They utilized local pH changes caused by an electrochemical reaction by synthesizing drug-loaded nanofilms, and the pH change produced by the electrical signal recovered quickly after the removal of the stimulus owing to a buffering action, which prevented an “off” state drug release [77].

## 4. Dual and Multiple Response Systems

In addition to the above single stimulus-responsive nanoparticles, combined triggers that use magnetic fields and pH, temperature and redox potential, temperature and enzyme presence, temperature and pH, and redox potential and pH combined with temperature and redox potential can be considered for smart-responsive drug-delivery systems [78,79,80,81]. The construction of these multi-stimuli-responsive nanoparticles would effectively regulate their precise release at target locations [82,83].

### 4.1. Temperature and pH Dual-Stimuli-Responsive Systems

Nanoparticles triggered by pH and temperature are the most widely investigated dual-sensitive nanosized smart-targeting nanotherapeutics. Various polymers that are both pH- and temperature-responsive have gained considerable interest in the last decade as carriers of chemotherapeutic drugs because tumor tissues have a lower pH and a higher temperature than healthy tissues. The most commonly studied thermo-responsive polymer is poly (*N*-isopropyl acrylamide) (pNIPAAm) because its LCST is approximately 32 °C, and its gel structure collapses above the LCST; thus, at body temperature (37 °C), the hydrogel shrinks. Combinations of pNIPAAm with pH-responsive polymers, such as polyacrylic acid and polyacrylamide, create dual-responsiveness [84].

Temperature- and pH-responsive nanocapsules consisting of pNIPAAm copolymers with glycyrrhetinic acid and acrylic acid were synthesized via the double-emulsion solvent evaporation by He et al. and were used to deliver DOX to tumor sites [85]. This study reported responses of different cell types, such as human hepatocytes (HL-7702) and hepatoma cells (HepG2) co-cultured with nanocarriers. They observed a significant reduction in HepG2 viability when treated with DOX-releasing nanocapsules. A higher temperature accelerated the molecular thermodynamic movement of DOX and, consequently, led to a faster drug diffusion rate [85].

Dual-stimuli-(pH and temperature)-sensitive nanosystems can be adopted for the transdermal drug-delivery applications since they can penetrate the deeper layers of skin tissues. Yamazaki et al. studied the effect of liposomal drug delivery on melanocytes via the transdermal route. Owing to the deep penetration, the modified liposomes delivered antioxidants or UV-protective agents to melanocytes residing in deep skin tissues [86].

Chen et al. prepared dual-stimuli-responsive microcapsules with drug loading and controlled release behaviors [87]. In their study, temperature-sensitive polymer particles (pNIPAM particles) were synthesized and loaded with Nile Red (NR) and oil-soluble fluorescent green (OG). At low pH conditions, the electrostatic repulsion between the positively charged polymer chains in the microcapsule shell caused the microcapsule shell to swell and release the OG, but the NR remained encapsulated inside the microcapsules. Therefore, the pH stimulus only triggered the release of OG molecules but did not have any influence on the NR molecules inside the particles. When the microcapsules were exposed to increasing temperatures, the NR molecules inside the particles were released because the microcapsules collapsed, but the OG molecules were unaffected [87].

### 4.2. pH and Redox Potential Dual-Stimuli-Responsive Systems

**Figure 2:**
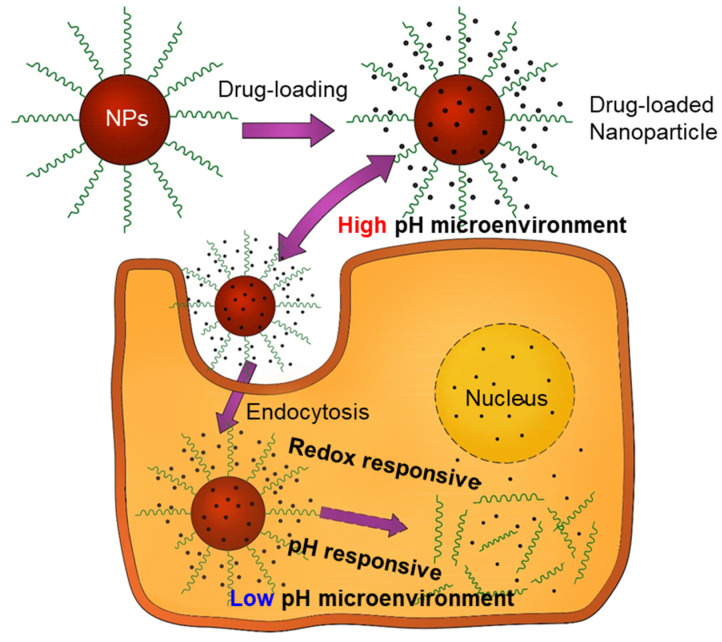
Schematic illustration of pH/redox potential dual-response-mediated drug release for tumor treatment. Black dots represent the drug molecules. Drugs are encapsulated inside the nanoparticles (red) and also adsorbed on the surface of nanoparticles (NPs). When endocytosis occurs, and nanoparticles are responding to a stimulus such as pH or redox potential, this may promote a drug release inside the cell.

Two of the most interesting intrinsic triggers are pH and redox potential because certain dysfunctional conditions such as cancer can locally alter them (Figure 2). Cancer cells contain high amounts of GSH (a tripeptide-containing cysteine), which is an important antioxidant with a high reducing capacity. GSH can undergo redox reactions, break disulfide bonds, and activate the release of drugs in cancer cells [88]. Therefore, drugs entrapped in structures containing disulfide bonds can dissociate more easily in cancer cells than in the rest of the body. Physically adsorbed drugs on the surface of nanoparticles also could be delivered to the target cells through an endocytosis, although those may not be as stable as drugs encapsulated in the NPs.

Tang et al. fabricated dual-sensitive and biodegradable core-crosslinked HPMA (*N*-(2-hydroxypropyl) methacrylamide) copolymer-doxorubicin conjugate-based nanoparticles [89]. A higher cumulative drug release (about 80.2%) was observed in the presence of GSH at a pH of 5.0 than in the presence of PBS (pH 7.4) without GSH (approximately 27.0%, 24 h). The cross-linked copolymer-NPs exhibited a strong in vivo anticancer efficacy in the A549 lung tumor model, as indicated by a high tumor inhibition rate (77.75%) [89].

Gong et al. prepared dual-responsive, DOX-containing micelles that have disulfide bonds linking PEG chains in the outer regions of the micelles [90]. At the tumor site, the PEG chains were released, destroying the integrity of the micelle and releasing DOX into the surrounding environment. Some results showed that at a pH of 7.4 without GSH, less than 5% DOX was released from the micelles after 176 h, mostly owing to strong hydrophobic and π-π interactions between the drug and the micelle cores. A low pH and high redox potential are favorable for DOX release from these micelles [90].

Ding et al. produced a redox potential/pH dual-stimuli-responsive drug-delivery system based on the amphiphilic self-assembly of hydrophilic polyacrylic acid (PAA-pH-responsive segments) and hydrophobic tocopherol succinate (TOS) [91]. A vesicle drug-delivery platform was constructed via the self-assembly of amphiphilic PAA-cystamine (cys)-TOS in an aqueous solution of methotrexate, an anticancer drug. Some results showed that more methotrexate was released with dual-stimuli (i.e., pH and GSH) than with a single pH stimulus because the disulfide bond of cystamine is sensitive to GSH and the amide bonds in PAA-cys-TOS are sensitive to pH, resulting in the collapse of the vesicles and accelerated drug delivery [91].

### 4.3. Dual-Stimuli-Responsive Systems

Light is an external trigger with the energy to cause precise changes in the organization of the functional groups or dielectric constants of polymers sensitive to electromagnetic radiation.

Li et al. prepared controlled-release nanoparticles (CDDP—cisplatin) that can reduce water infiltration and enhance the nanocarrier’s stability and half-life in plasma [91]. After irradiation with NIR, a certain amount of ROS is produced. The ROS readily oxidizes the tellurium because of its low electronegativity, which weakens the interaction of tellurium and CDDP. This caused the rapid release of CDDP from the system, and tellurium was oxidized at the tumor site. The results showed that, within 10 min, most of the CDDP in the nanoparticles was released upon laser exposure (808 nm). This is caused by the core-shell multilayer structure generated from the oxidation of tellurium on the polymer backbone by the O_2_ produced by ICG. The core expanded gradually, but the shell shrank as the oxidation process increased, and the size of the Pt-ICG NPs became larger after NIR irradiation. The oxidation of tellurium made the hydrophobic block of the polymer more hydrophilic, which facilitated the free diffusion of water in and out of the nanoparticle core. This chemical property and the structural changes enable increased CDDP release from the nanoparticles [92].

Redox potential and light dual-stimuli-responsive systems are advantageous in cancer therapy and targeting applications. NIR light, as a stimulating factor, has been widely studied by researchers. Photothermal therapy based on photothermal conversion agents that can convert NIR light to heat can inhibit tumor cells. It is worth mentioning that a photothermal treatment is based on external NIR light that can deeply penetrate human tissues without invasive damage.

You et al. created ICG- and CDDP-loaded smart nanoparticles that were NIR- and redox potential-responsive (i.e., exogenous and endogenous stimuli-responsive) to target tumors [93]. The presence of GSH can break disulfide linkages and damage the cross-linked core structure. Conversely, increased temperature via NIR radiation can result in more significant destruction of the nanoparticle lipid bilayer. Thus, the redox potential and NIR radiation dual-stimuli-responsive nanoparticles exhibit excellent smart-release characteristics. Therefore, the combination of NIR light and GSH stimuli-responsive drug release demonstrated considerable potential as a vehicle for the controlled release of drugs [93].

### 4.4. Magnetic Field-Responsive Delivery System

Magnetic properties and magnetic field intensity should be considered when designing magnetic field-responsive delivery systems. Magnetic nanoparticles, particularly Fe_3_O_4_ particles, can be combined with pH- or redox potential-responsive materials to construct multi-stimuli-responsive delivery systems that can control the drug release.

Zhang et al. prepared a magnetic/pH dual-responsive nanocomposite via the amide condensation of PEGylated citric-coated Fe_3_O_4_ (PCI)-graphene oxide (GO) nanocomposites [94]. The DOX-loaded PCI-GO nanocomposites exhibited a pH-responsive release behavior. The study used solutions with pH values of 5.5 and 7.4 to demonstrate that the release rate increased under an acidic environment. Under acidic conditions, the amine group on DOX was protonated and could not participate in the formation of a hydrogen bond. This weakened the bond between DOX and PCI-GO and accelerated the release rate [94]. Therefore, PCI-GO nanocomposites can be used as a magnetic drug-delivery system for therapy.

Another study assembled folic acid onto chitosan [95]. Oleic acid-modified Fe_3_O_4_ nanoparticles, hydrophobic drugs, and green fluorescent dyes were encapsulated in microcapsules (approximately 500 nm) targeted by a red fluorescent dye to construct a chitosan-based microcapsule with both redox potential and magnetic field-responsiveness. The results indicate that the loaded drug was released slowly in the presence of GSH and a magnetic field.

## 5. Multi-Responsive Systems

Systems sensitive to more than two stimuli are becoming increasingly attractive. When systems with more than two sensitivities, such as pH, temperature, and redox potential, magnetic fields, and temperature, are combined in the same matrix, they are called multi-responsive systems [96,97,98]. These systems play a vital role in nanomedicine because they act as smart carriers that release their payload on demand. These multi-responsive nanoparticle systems possess all the advantages of the individual stimulus approaches in addition to precise control over drug delivery to pathological sites. Multi-responsive designs not only respond to an individual stimulus for drug targeting, but they also display enhanced release when simultaneously triggered by multiple stimuli.

### 5.1. Magnetic Field, Temperature, and Redox Potential Multifunctionality

Multi-responsive nanocarriers that are sensitive to temperature and redox potential can effectively release their drug payload in the tumor microenvironment. Hegazy et al. reported magnetic mesoporous silica nanoparticles (MMSNPs) that have iron oxide nanoparticles as a core inside a mesoporous silica coating [99]. They can be used in a wide range of disciplines and provide promising results. These magnetic cores release their drug payloads via non-invasive and highly penetrating alternating magnetic fields, which can cause localized heating via magnetic losses of the nanoparticles.

Through in vitro loading and the triggered release of DOX, some results indicate that MMSNPs-PNIPAAm NPs exhibit both a high drug-loading capacity (19.6%) and encapsulation efficiency (73.8%) and successfully encapsulate DOX based on an absorbance peak at 480 nm. In terms of a temperature-triggered DOX release, this result shows that thermo-sensitive nanocarriers retain their drug payload at room temperature and rapidly release it near a locally-heated tumor (40–42 °C) to counteract blood-passage time and washout from the tumor.

Regarding redox potential stimuli-responsiveness, the addition of tris(2-carboxyethyl)phosphine (TCEP; a reducing agent that causes the cleavage of disulfide bonds) causes rapid aggregation into macroparticles in water. In the presence of TCEP, a drug release from MMSNPs-PNIPAAm/DOX NPs reached 45% compared to less than 20% without TCEP over 24 h. It was concluded that the drug release from the nanoparticles was responsive to a reducing environment owing to the cleavage of disulfide bonds. PNIPAAm/DOX NPs exposed to a magnetic field were able to release significantly more DOX than the NPs exposed to a temperature below the LCST (25 °C) at the same time. Other results indicated that both a magnetic field and a high temperature enhanced DOX release over time and reached 86% after 24 h, applying reducing potential and temperature together resulted in an 80% release. In summary, simultaneously applying thermal, redox potential, and magnetic-sensitive environments significantly improved the drug release from PNIPAAm/DOx NPs that reached 98% within 24 h [99]. The amount of drug release triggered by a random combination of two or three stimuli was higher than that mediated by any of the stimuli separately.

### 5.2. pH, Light, and Enzyme Multifunctionality

Various multifunctionalities, including pH, light, and enzyme stimuli-responsiveness, can be added to nano-drug-delivery systems to produce theranostics that can detect tumors and release drugs at the tumor site.

Copper sulfide nanoparticles (CuS NPs), which are composed of NIR-resonant materials, can be used in theranostics [100]. CuS is a photosensitizer that can be used in photothermal therapy. It generates cytotoxic ROS to induce photodynamic therapy under NIR exposure. Feng et al. synthesized hollow mesoporous CuS NPs coated with HA (hyaluronidase), which is an extracellular matrix substance with an expanded random coil structure, and loaded them with DOX for tumor treatment [101]. HA helps minimize a premature drug release by coating the surface of nanoparticles.

CuS produces heat upon exposure to NIR, which is used as hyperthermic phototherapy against cancerous cells. A drug release is controlled by the presence of HA and an enzyme that cleaves HA inside the liposomes. In vitro experiments showed that only small amounts of DOX are released in an acidic environment and under NIR irradiation. In the presence of HA, the DOX release increased 26.6% from a pH of 7.4 to a pH of 4.5 within 14 h, and the DOX release was approximately 39.7% under NIR irradiation. This indicates that the enzymatic degradation of the HA coating facilitated DOX diffusion from the hollow interior into the surrounding medium. Under NIR irradiation, the DOX release was further enhanced; this may be caused by the heat produced by hollow mesoporous CuS, which decreases the viscosity of the surrounding fluid and facilitates drug diffusion [101].

## 6. Challenges and Strategies

Nanomaterials are advantageous in numerous biomedical applications due to their potential benefits with high drug loading, surface chemistry, biodegradability, and drug release characteristics accompanied by stimuli-sensitives such as light, temperature, ultrasound, etc. However, some limitations include toxicity, biocompatibility with certain types of cell membranes, or the size/shape of nanocarriers that need to be solved to optimize the efficiency of drug release in chemotherapy applications for safety purposes. In order to overcome the toxicity or cytotoxicity of nanomaterials that are harmful to humans, some approaches are proposed to develop compatible nanomaterials such as by surface modification with different biodegradable molecules. For example, Malvindi et al. [102] prepared bare SPIONs and thin silica shell-coated SPIONs (Fe_3_O_4_/SiO_2_ NPs) and conducted an in vitro experiment on A549 and Hela cells to probe the cytotoxicity. The results showed that the Fe_3_O_4_NP coated surface could minimize the oxidative stress and iron homeostasis alteration and thus reduce the overall toxicity. From their study, it can be observed that Fe_3_O_4_/SiO_2_ NPs are efficient platforms for preventing dissolution, hindering the toxic ions, and enhancing the stability of the particles in the reduction of cytotoxic effects [102]. Finally, Woziak et al. [103] compared the various morphologies of AuNPs, including nanospheres, nanowires, nanostars, nanoflowers, and nanoprisms in term of cytotoxicity aspects, indicating that toxicity can be distinctly affected by the shape of nanoparticles.

The results collected from testing analyzed nanoparticles in the range of 1–300 µM showed that the lowest cytotoxicity could be seen in spherical nanoparticles. Another work carried by Reznickova et al. [104] evaluated the role of functional groups to the cytotoxicity of nanomaterials. They examined the effect of nanoparticles with unmodified PEG, polyethylene glycol (PEG), PEG-SH, and amino (PEG-NH_2_) groups. AuNPs with PEG-SH showed no cytotoxicity compared with PEG and PEG-NH_2_ nanoparticles.

Biodegradability is another essential factor that needs to be considered because it affects the stability of drug-carrier particles in the biological medium. Some biodegradable nanomaterials such as PLGA micelles, PCL nanoparticles, chitosan nanoparticles, dendrimers, and liposomes with their specific composition and administration strategy are utilized to improve therapeutic effects and to decrease side-effects [105]. For instance, Parmar et al. [106] produced PLGA nanoparticles encapsulating methotrexate-transferrin to enhance the biocompatibility of cell membranes. Folic acid as a ligand was utilized to function with PLGA nanoparticles for the absorption across the blood-brain barrier. In another study, Christos Tapeinos et al. [107] used biodegradable PLGA microspheres coated collagen Type I and MnO_2_ particles to protect cells under harsh oxidative conditions. Therefore, nanomaterials with biodegradability hold a promising future in nanomedicine for overcoming problems associated with biocompatibility properties.

## 7. Conclusions

Recently, wide-ranging applications of nanotechnology and nanomaterials have made profound impacts on the pharmaceutical industry and, consequently, give rise to nanomedicine. The primary features of stimuli-responsive nanoparticles applied in biomedical applications, especially drug delivery, have attracted the interest of many researchers owing to their high loading capacity, biocompatibility, and stimuli-responsive drug release characteristics. Such characteristics also provide a promising avenue to enhance the effectiveness of traditional drugs and promote the rapid development of disease diagnostics and treatments.

Despite significant advancements being made with nanomaterials in the biomedical or pharmaceutical areas at large, or specifically regarding drug delivery, many limitations remain. Biocompatibility, biodegradability, lower toxicity, and safer elimination of smart nanomaterials are some of the obstacles that need to be considered before designing a nanomaterial-based drug-delivery system. Additionally, the size of the carrier is another important feature that requires consideration, and administrative issues involved in using polymeric-based smart drug-delivery systems are another major drawback in this field. To solve these problems, future investigations should explore and design drug-delivery systems that use novel polymeric materials that are biocompatible and non-toxic.

**Figure 3:**
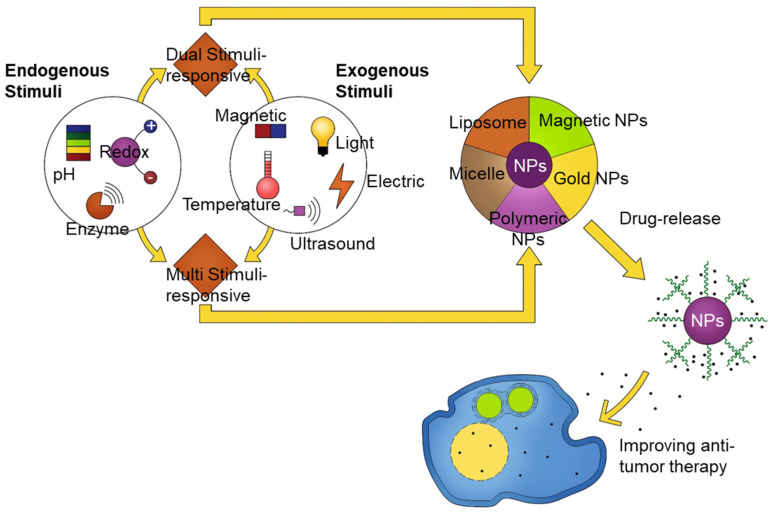
Scheme showing the future perspectives of combination stimuli-sensitivities and nanomaterials for biomedical applications.

From a long-term perspective, dual- and multi-stimuli-responsive nanomaterials (e.g., pH and redox potential dual stimuli; temperature and pH dual stimuli; pH, light, and enzyme presence multi stimuli) may pave the way for unique nanosystems that are beneficial in biomedical applications (Figure 3).
